# Amateur Runners’ Commitment: An Analysis of Sociodemographic and Sports Habit Profiles

**DOI:** 10.3390/ijerph17030925

**Published:** 2020-02-02

**Authors:** David Parra-Camacho, Manuel Alonso Dos Santos, María Huertas González-Serrano

**Affiliations:** 1 Department of Physical Education and Sports, Faculty of Physical Activity and Sport Sciences, Universitat de València, 46010 Valencia, Spain; david.parra-camacho@uv.es; 2 Department of Management, Faculty of Economics and Business Administration, Universidad Católica de la Santísima Concepción, Concepción 4090541, Chile; malonso@ucsc.cl; 3 Department of Teaching and Learning of Physical Education, Plastic and Music Education, Universidad Católica de Valencia, 46110 Valencia, Spain

**Keywords:** runners, commitment to running, clusters, sports habits, running addiction, urban runners, amateur runners

## Abstract

The aim of this work is to analyse the commitment to running among urban runners by identifying groups regarding commitment to this sport and by defining their sociodemographic profile and their sports habits. A sample of 1806 participants in popular urban races in the city of Valencia was interviewed using an 11-item questionnaire on commitment to running, sociodemographic characteristics, and sports habits. The psychometric properties of the running-commitment scale allowed for the identification of two factors in commitment to running: enthusiasm for running (6 items) and affliction from running (5 items). Subsequently, a cluster analysis combining hierarchical and non-hierarchical methods was performed, identifying three groups of runners: highly committed (n = 650), moderately committed (n = 749), and slightly committed (n = 407). Highly committed runners positively rate all aspects of running enthusiasm (M = 4.15), while moderately committed runners show a more neutral attitude (M = 3.41) and slightly committed runners disagree on these aspects (M = 2.41). Both highly (M = 1.32) and moderately (M = 2.04) committed runners disagree on the affliction-related aspects of running, while slightly committed runners show a trend towards neutrality on some affliction indicators. The variables referring to age, level of studies, sports habits, and running addiction contributed to differentiating the identified groups.

## 1. Introduction

Today, running has become an immensely popular pastime practised in the public sphere by millions of recreational participants around the world. However, until the 1960s, recreational jogging in streets, parks, or forests was considered an odd activity [1]. An example of the boom in running that has taken place in some Spanish cities can be found in Valencia (Spain), where currently more than 30 popular races are organized every year [2]. Valencia is a city with a strong running tradition, with runners participating in this activity since the beginning of the 19th century [2]. Also, the number of nonprofessional sports clubs offering different sports activities has been increasing recently in the main European cities [3,4]. However, both nonprofessional and professional clubs have limited information about the profiles of athletes based on their commitment to the activities they offer. The groups of runners are heterogeneous and have different subcultures; they could be differentiated in the frequency of the practice of the sport, in the motives, or in the participation in competitions [5]. For each group, sports associations and clubs and governmental or nonprofit organizations can design communications, activities, and services specifically targeted at them. Commitment could be a segmentation variable to obtain different groups of subjects with different motivations, activities, and interests.

Commitment is a psychological construct that, from a sporting point of view, represents the desire and willingness to continue practising a sport [6,7]. When associated with positive factors, such as the intrinsic enjoyment of the activity itself; opportunities to participate successfully; personal investments of time, money, and experience in the sport; and social pressures from parents, coaches, peers, etc., commitment increases in tandem with increases in these factors [8]. However, when associated with negative factors, such as alternatives for successful participation in other attractive sports, commitment decreases [8].

Another concept that has been associated with or related to this construct in previous research on commitment to running is the notion of a positive addiction to running, defined by Glasser [9] as enjoyment that increases mental strength and, when lost, produces some type of physical or psychological discomfort [10]. This concept is opposed to that of a negative addiction to continuous running, which can dominate the runner’s life and can produce the unwanted effects of overtraining syndrome [11]: fatigue, decreased performance, and mood disorders.

Some studies have observed among endurance sports participants a relationship between commitment to and dependence on exercise [12]. Endurance athletes usually train a considerable number of hours, which some studies have found to have a positive correlation with addiction to running among marathon runners [13,14]. However, most research indicates that commitment to running and negative addiction to running are two different concepts predicted by different variables and that, although there is a high correlation between them, the two phenomena do not necessarily appear together [15,16,17,18,19].

In this regard, Pargman [20] built on these concepts (positive and negative addiction to running) to describe the existence of two types of runners: addict-dependent runners, who have a positive addiction towards continuous running and perceive joy and happiness in running (if they do not do it, they feel bad), and committed-dedicated runners, who have a negative addiction towards continuous running and a broader, rational, and pragmatic intellectual component that makes them give continuous running a very high priority in their lives without necessarily liking it.

Among the first contributions on commitment to running, Carmack and Martens [21], who developed and validated the unidimensional commitment to running (CR) scale, equate commitment to running with the concept of positive addiction to continuous running. Subsequently, several studies have used this scale to analyse the characteristics of runners and to learn about their sociodemographic profile and sports habits. Carmack and Martens [21], and Joseph and Robbins [22] explained that the variables that contributed to increasing the commitment to running were related to the time spent on training (number of days of training/week and minutes/training), while Thornton and Scott [23] also highlighted distance covered in kilometres as a predictor variable. Improved personal performance among marathon runners was an important factor in increasing the commitment to running for this profile of runners, while amateur runners emphasized the intrinsic enjoyment of the activity or improved health [24].

In the Spanish context, several investigations have also been carried out among Spanish runners on the psychological variables related to motivation, commitment, and addiction to running [5,19,25,26,27]. In this regard, it is important to highlight the validation of the Carmack and Martens scale [21] in Spanish marathon runners by Ruiz-Juan and Zarauz [25], who retained 11 indicators from the 12 proposed on the original unidimensional scale that allowed for evaluation of the commitment of the runners. Several studies have analysed the relationships between training variables and sports habits and the commitment and addiction to running in marathon runners [18,19,28]. In addition, the motivation of amateur Spanish runners when participating in this physical activity has also been analysed in various studies [5,26,27].

However, studies that segment runners into groups with different behaviours towards running are not abundant, and most of the contributions in this regard have focused on identifying groups or on classifying runners according to their motivation to practice this physical activity [5,29,30] rather than according to their commitment to running. 

Therefore, numerous contributions that analyse diverse psychological variables such as runners’ motivation [17,20,26,27,30,31,32,33,34,35], addiction [15,19,28,36], or commitment [12,19,21,22,23,25,37] to running have been made in the study of the running phenomenon. However, many of these studies have focused on long-distance runners, such as half-marathon and marathon runners [19,28,31]. There are few contributions that focus on Spanish urban runners who participate in short-distance races [5,26,27] and that classify runners into clusters according to their commitment to running, identifying groups with different sociodemographic characteristics and sports habits. Research on short-distance (versus long-distance) races allows researchers to study a larger universe because more and more people practice sports for health and social reasons but are less concerned about winning long-distance races [38].

This paper analyses the commitment to running among runners who participated in urban races of less than 10 km. These data provide information on the behaviour of this type of urban sportspeople in the context of Spanish short-distance runners. Spain is a country with an established running practice: the percentage of the population who practice running is 30.4% [39].

The aim of this paper is to analyse the commitment to running among urban runners by identifying groups with a greater or lesser commitment to this sport. In addition, the profile of these groups is defined to determine their sociodemographic characteristics and to be able to analyse their sports habits.

## 2. Materials and Methods

### 2.1. Sample, Procedures, and Questionnaire

In this study, a sample of 1806 runners who participated in the city of Valencia’s urban circuit of popular races during 2015 were interviewed. The circuit has 10 races. The total number of runners in the circuit was 50,038 recurring runners (M = 5004). The sample size used is at least 95% confidence (5% margin of error). The questionnaires were collected after the end of this race circuit during the month of January 2016 by using an online format. This research used a self-supplied online questionnaire because of the difficulty of obtaining valid responses among active or tired athletes immediately after the race. 

The questionnaire was made up of three sections of questions. The first included questions of a sociodemographic nature referring to age, gender, occupation, level of studies, and income.

The second section contained questions referring to the sports habits of the participants: number of years they have been running, weekly frequency with which they run, preferred distance when participating in a popular race, affiliation with a club, whether the club is federated, with whom they usually run, the distance they usually run weekly, the athletic level they consider themselves to have as runners, the number of long-distance races finished (half marathons and marathons), future intentions regarding participation in urban races, and three items on addiction to running. The three indicators on the intentions of amateur runners were adapted from the scale on behavioural intentions by Zeithaml, Berry, and Parasuraman [40], whereas the three indicators on running addiction were extracted from the Spanish validation by Zarauz and Ruiz-Juan [36] of the scale on running addiction. The adaptation of the scale consisted in translating and adapting the scales to the race under research. The indicators on behavioural intention of amateur runners showed adequate reliability (Cronbach alpha = 0.91, Composite Reliability = 0.92; Average Variance Extracted = 0.79). The indicators of both future intentions and running addiction were assessed with a five-point Likert scale with the following response options: 1 = strongly disagree, 2 = disagree, 3 = neither disagree nor agree, 4 = agree, and 5 = strongly agree.

The third section includes indicators related to the participants’ commitment to running. The Ruiz-Juan and Zarauz scale [25] validated in the Spanish context in marathon runners was used to collect participants’ perceptions of their own commitment to running. This scale is composed of 11 indicators evaluated by the interviewees through a Likert scale with the same response options as those of future intentions and running addiction. 

For this type of research, it was not necessary to get approval from the Committee of Ethics of the university where this study was carried out (University of Valencia). According to the Committee of Ethics and Human Research from this university, it is not necessary to get approval to carry out an opinion survey about a topic or issue, professional status, or satisfaction with certain matters.

However, it is obligatory to include a preamble in the survey with presented information about the project (theme and purpose), the benefits that the information collected by the survey may provide, the willingness of the participation, and the anonymous treatment of data (Data Protection Law). It is also compulsory to indicate a contact person to ask for further information and to put one paragraph in which the survey respondent voluntarily accepts participation in the research and gives consent tacitly when responding to the survey. Thus, following these guidelines indicated by the Committee of Ethics and Human Research from the University of Valencia to develop this sort of research, all this information was added at the beginning of the questionnaire.

### 2.2. Statistical Analysis

First, the psychometric properties of the running commitment scale were tested on the sample under study by performing an exploratory factor analysis (EFA) and a confirmatory factor analysis (CFA). The EFA was performed with the FACTOR program following the recommendations of Lloret-Segura et al. [41] using the maximum likelihood (ML) method and oblimin direct rotation. To determine the number of factors, the optimal implementation of the parallel analysis procedure was used [42]. The model fit was observed using the root mean square of the residuals (RMSR) coefficient as well as the goodness-of-fit index (GFI) proposed by Tanaka and Huba [43]. The RMSR should be less than 0.05 [44], and the GFI value should be less than 0.95 [45]. Other indicators that were taken into account were the generalized G-H index (>0.80) to analyse the replicability of the factors derived from the EFA [46]. The measures for sample adequation of Kaiser Meyer Olkin (KMO) were also observed, as was Bartlett’s sphericity test. Items with factorial loads below 0.40 or above this value on two or more factors were eliminated before the next EFA was carried out.

After applying the EFA, a CFA was performed on the factorial solution derived from the EFA using robust maximum likelihood estimation (MLR) with the aim of correcting the possible absence of multivariant normality using statistics such as the χ^2^ of Satorra-Bentler [47]. Thus, for the evaluation of global fit, different goodness-of-fit indices recommended in the literature [48], such as the significance of the chi-squared value and its robust correction offered by Satorra-Bentler (S-B χ²) [49], were used. In addition, other coefficients that allowed for testing the adequacy of the proposed models, such as the ratio of χ² and its degrees of freedom (χ^2^/df) [50], were calculated, with the acceptable values being less than five [51]. However, these indices are affected by the sample size, so the standardized root mean square residual (SRMR) index was used, where values of 0.06 or less indicate an excellent fit and values of 0.08 or less indicate a good fit [51]. In the same way, the coefficients of the indices of robust goodness-of-fit of the proposed model, the compared fit index (CFI) and the incremental fit index (IFI), were tested. For these indicators, a fit with values above 0.90 is considered good [52]. To finalize, the root mean square error of approximation (RMSEA) is shown, with a score below 0.08 being considered a good fit [53].

In the evaluation of the reliability of the scale, three measurements were taken into account: the Cronbach’s alpha, composite reliability (CR), and average variance extracted (AVE) values for each factor [54]. In addition, convergent validity was tested through the significance of the factorial loads in their respective dimensions and the values of the associated *t*-tests. Additionally, discriminant validity, which has to do with seeing the clear distinction between any pair of constructs, was evaluated using the method suggested by Fornell and Larcker [55]. This method confirms discriminant validity if the square root of the AVE value of a determined factor is greater than the correlation coefficients between the factor and any other in the proposed scale. The other criterion to assure discriminant validity is that the correlations between the different pairs of factors must be less than 0.85 [48].

After checking the psychometric properties of the running commitment scale, a cluster analysis was performed to identify groups of participants with different characteristics according to their running commitment. This analysis was performed using the statistical program SPSS version 24.0 for Windows (IBM, Armonk, NY, USA), with the 11 items of the running commitment scale. Two estimation methods (hierarchical and nonhierarchical) of the cluster solution were combined to optimize the results. The hierarchical cluster analysis was performed using the Ward clustering process and, as a measure of similarity, the Euclidean distance squared. A nonhierarchical analysis was applied to the groups proposed in the previous analysis through the K-means method using as initial centres the means of the variables obtained for each cluster solution of the hierarchical analysis. To define the characteristics of the group profiles and to evaluate predictive validity, ANOVAs and chi-square tests were performed with variables that were not included in the initial analysis. The value of the contingency coefficient (C) was also used to check the intensity of the association and the size of the effect of the related variables.

## 3. Results

### 3.1. Sociodemographic Characteristics

Table 1 shows the sociodemographic characteristics. The respondents had an average age of 39.48 (SD = 9.21), 74.4% being men and 25.6% being women. In terms of occupation, most are employed (88.4%). The majority was university studies (59.2%) and had an income level of less than 18,000 euros per year (51.3%).

### 3.2. Psychometric Properties of the Commitment to Running Scale

First, the properties of the items on the commitment-to-running scale were analysed by checking the corrected correlation item–total values, as well as the mean, standard deviation, asymmetry, and kurtosis values. Table 2 shows the statistics. The mean scores of five indicators were inverted as indicated by Ruiz-Juan and Zarauz [25]. The values of the item–total corrected correlation coefficients were higher than the cutoff point recommended by the literature (≥0.30) [56]. Additionally, the values of asymmetry and kurtosis are acceptable for most variables because they are lower than 3.0 [57] except for item CR7, of which the value exceeded the recommended cutoff point for ensuring a normal distribution of the data. On the other hand, indicators on the intentions of amateur runners showed adequate reliability with a Cronbach alpha of 0.91, CR = 0.92, and AVE = 0.79.

After checking the properties of the items, an EFA was carried out for the 11 items related to the running commitment of the interviewed participants. The parallel analysis procedure suggested grouping the indicators into two factors. It was not necessary to eliminate any indicators because all of them had factor loads above 0.40 and no cross-factor loads exceeded this value in the two factors. Table 3 shows the grouping of the indicators into the two factors: enthusiasm for running (6 items) and affliction from running (5 items).

To check the fit of the model, the RMSR and gamma index or GFI coefficients were analysed, which showed values within the recommended cutoff points: RMSR = 0.03 (<0.05) and GFI = 0.99 (>0.95). In addition, the generalized G-H index showed values higher than 0.80 in the two factors detected by the EFA (0.85 for the running enthusiasm factor and 0.80 for the running affliction factor), indicating the possibility of good replicability of the dimensions in other studies [46]. The variance explained by the 11 items grouped in the two factors was 54.78%.

On the other hand, a CFA was derived from the two-factor solution of the EFA and from the unidimensional proposal of the scale presented by the authors of the original scale [21]. The CFA considering the scale as unidimensional did not show a good fit, as seen in the goodness-of-fit indices (χ^2^ = 2005.81 (df = 44); *p* < 0.01; SRMR = 0.125; RMSEA = 0.122 (confidence interval (CI) = 0.116–0.128); CFI = 0.73; IFI = 0.73)).

However, the factorial solution derived from the EFA by grouping the indicators into two factors did show optimal model fit values. The value of χ^2^ was significant (χ^2^ = 653.95; df = 43; *p* < 0.05), although the relationship between the value of χ^2^ and the degrees of freedom (normed chi-square) was quite high since values below five are considered acceptable [51]. This is because the value of this statistic is very sensitive to the size of the sample, which could erroneously indicate a poor adjustment of the data [48,58]. For this reason, it is recommended that other goodness-of-fit indices be used [48]: SRMR, RMSEA, CFI, and IFI. These indicators showed a good fit: (SRMR = 0.077; RMSEA = 0.076 (IC = 0.070–0.082); CFI = 0.90; IFI = 0.90)).

To analyse reliability, the Cronbach’s alpha, CR, and AVE measurements were observed (see Table 4). The Cronbach’s alpha values were higher than the 0.70 recommended by the literature [54]. This criterion was also fulfilled for the CR values [55], with values of 0.83 for the running enthusiasm factor and 0.77 for the running affliction factor. Finally, for the AVE values, it was found that the two factors did not have values higher than the 0.50 recommended by the literature [59]. According to Hatcher [60], when the reliability of the construct is acceptable, a marginally low AVE value can be accepted (see Table 4). Therefore, the decision was made to retain these two factors without combining them into a single factor since the unidimensional solution did not offer a good fit.

To analyse convergent validity, it was found that the values of the *t*-tests associated with the factorial loads of the items were higher than 1.96 (*p* < 0.05), ranging from 15.06 to 27.57. Regarding discriminant validity, on the one hand, the correlation between the two factors was less than 0.85 (r = 0.35; *p* < 0.01). On the other hand, it was found that the square root of the AVE value was higher than the correlations between pairs of factors. This criterion was also complied because the values of the square root of the AVE were √AVE = 0.67 for factor 1 and √AVE = 0.63 for factor 2.

### 3.3. Identification and Description of Clusters

A cluster analysis was conducted to identify groups with different characteristics according to their commitment to running among the interviewed runners participating in urban short-distance races. Following the procedure recommended by Hair et al. [54], a hierarchical cluster analysis was first performed using Ward’s method of observing the increase in the agglomeration coefficients between clusters two and three, three and four, and four and five. After observing these coefficients, the solutions of two, three, four, and five groups were used to apply the second analysis of the k-median clusters by using the initial centres from the hierarchical cluster analysis. It was considered appropriate to contrast all the solutions because there are not many previous studies that identify clusters of amateur runners according to their commitment to running, as mentioned in the theoretical framework.

This study used the solution of three clusters because it identified three groups of urban runners with different levels of commitment to running, helping to interpret and identify their sociodemographic profiles and sports habits. It is important to note that the choice of an ideal cluster solution depends on the theoretical foundations, common sense, and practical judgement of the researcher [54]. Table 5 shows the mean values (centroids) for each of the 11 variables of the commitment-to-running scale introduced in the analysis and the results of the ANOVA test carried out to confirm the significance of the differences between groups (F statistic and significance level). In this table, it can be seen that all the mean scores of the identified groups present statistically significant differences (*p* < 0.001). The variables that distinguish the clusters most correspond to the running enthusiasm factor: “Running is vitally important to me” (F = 896.45) and “Life is so much richer as a result of running” (F = 768.53). The item that differentiates the clusters least is “I dread the thought of running” (F = 47.61).

Cluster 1 was labelled “highly committed” (n = 650; 35.99%) because all indicators related to Factor 1 about running enthusiasm present averages close to or above the value of four on the Likert scale, which indicates a high degree of agreement with these statements. This group of runners presents a high commitment to this physical activity because they consider running to be of vital importance for their lives (M = 4.08), they would reorganize or change their schedule to satisfy the need to run (M = 4.09), and they consider their lives to be much richer because they practice this physical activity (M = 4.12). In addition, they show a high degree of agreement about the desire to run (M = 4.42) and enjoy the experience of running (M = 4.51). However, they disagree with the aspects related to factor 2 about a sense of affliction from running. For example, they tend to disagree with items related to a sense of drudgery over the activity (M = 1.46), an absence of enjoyment when running (M = 1.16), a feeling of dread over the practice of running continuously (M = 1.06), and a sense of relief when not running for one day (M = 1.20).

Cluster 2 was identified as “moderately committed” (n = 749; 41.47%) and includes a higher proportion of the interviewed runners; this group of runners is characterized by presenting average scores with a tendency towards agreement on some indicators of the dimension of enthusiasm for running but with a more moderate tendency than in cluster 1. Thus, the runners in this group consider the practice of running to be a pleasant activity (M = 4.01) and are willing to practise it (M = 3.73). They also show a positive tendency in considering that running enriches their lives (M = 3.47). However, they do not consider the practice of this physical activity to be essential to their lives (M = 3.16) or to be the high point of their day (M = 2.83). On the other hand, this group also disagrees with most of the negative aspects (affliction for running) associated with a commitment to running. However, the indicator related to the need to force themselves to run shows a score close to the value of three on the scale (M = 2.65), which would indicate some neutrality in the assessments of this group.

Cluster 3 was labelled “slightly committed” (n = 407; 22.54%) because they show a tendency to disagree with many aspects related to enthusiasm for running. In this regard, they disagree with the items stating that the practice of this physical activity is of vital importance for them (M = 2.00), that their life is much richer because they practice running (M = 2.18), that the practice of this activity is the high point of their day (M = 1.87), and that they reorganize their schedules to satisfy the need to run (M = 2.09). This group only shows a tendency to agree that running is a pleasant activity (M = 3.58). In the same vein as the other groups, these runners show disagreement with the aspects related to a sense of affliction from running, although there is a tendency towards neutrality in the aspects referring to a sense of drudgery over the practice of this activity (M = 2.91) and a need to force themselves to run (M = 2.77).

### 3.4. Profile of the Groups

Table 6 describes the profile of the runners that make up each group using other independent variables (sociodemographic and sports habits variables) that allow us to ensure the predictive validity of the groups, the percentages for each sociodemographic, and sports habits variable according to the cluster.

Statistically significant differences were observed among the groups in the case of the sociodemographic variables related to age (F(2, 1803) = 5.73, *p* < 0.05) and level of studies (χ^2^(6) = 18.94, *p* < 0.01), although the size of the effects (contingency coefficients) presented reduced values (see Table 6). 

It was also found that there were statistically significant differences in the mean scores among the groups on the indicators related to running addiction: “Some days, even if I do not feel like running, I do it anyway” (F(2, 1803) = 104.78, *p* ≤ 0.001), “I feel like I need to run at least once every day” (F(2, 1803) = 150.05, *p* ≤ 0.001), and “I’ve stopped running for at least a week for some other reason than injury” (F(2, 1803) = 46.84, *p* ≤ 0.001). Additionally, on the indicator related to future intentions to recommend participation in popular running races to others, statistically significant differences in mean group scores were observed (F(2.1803) = 4.72, *p* < 0.01).

The highly committed group is characterized by an average age of 39.51 (SD = 9.33), with a majority percentage of men (76.62%), which is higher than the other groups. It is verified that there is a greater degree of agreement in this group with indicators related to running addiction, as they show a tendency towards agreement on, for instance, the item stating that, some days, even if they do not feel like running, they go running anyway (M = 3.71), with a significantly higher average compared to the other two groups. Significantly higher differences, albeit with a more neutral trend in the average scores, are also observed in this group for the indicator related to the need to run at least once every day (M = 3.04) and lower differences for the indicator related to having stopped running for at least one week for a reason that was not injury related (M = 3.04). Finally, the runners in this group strongly agree with the items on their future intentions regarding participation, recommendation, and positive comments about running popular races, with slightly higher scores on all these indicators than those of the other two groups.

The moderately committed group is characterized by a higher average age of 40.13 (SD = 9.26), significantly higher than the slightly committed group, and a majority percentage of men (73.03%). These runners tend towards agreement on the indicator related to having stopped running for at least one week for a reason other than an injury (M = 3.58), with a significantly higher average compared to the higher commitment group level. In the same way as the other groups, they strongly agree with the items on their future intentions regarding participation, recommendation, and positive comments on popular races.

The third, slightly committed group is characterized by the lowest average age with 38.22 (SD = 8.79) and a majority percentage of men (73.22%). They tend towards agreement on the indicator related to having stopped running for at least one week for a reason that was not injury related (M = 3.75) with a significantly higher average compared to the group with greater commitment to the race. Following the trend observed in the other groups, they strongly agree on the item on their future intentions regarding participation, recommendation, and positive comments on popular races.

## 4. Discussion

This study analyses the running commitment of runners participating in urban popular races from the commitment-to-running scale validated with Spanish marathon runners. The analysis of the psychometric properties of the scale showed that the grouping of the indicators into two factors presented a better adjustment to the data collected in this study, identifying two dimensions related to positive aspects (enthusiasm for running) and negative aspects (affliction from running) of the psychological construct of commitment to running. In previous works on the validation of the running-commitment scale, the possibility of grouping the indicators under two factors was assessed but was rejected due to the lack of interpretability and the high degree of uncertainty of the second factor identified [25]. For this reason, it was decided to test the fit of the scale on a single factor, although the various goodness-of-fit indices showed values distant from those recommended in the literature as acceptable.

This study identified three groups of participants in urban popular races with different commitments to running: “highly committed”, “moderately committed”, and “slightly committed”. The denomination of these groups was made from the interpretation of the average scores of the variables on the commitment-to-running scale that allowed us to identify three groups with clearly different levels of commitment towards the practice of this physical activity.

The sociodemographic variables that contributed to significantly differentiating the identified groups were age and educational level. However, previous studies that have analysed the commitment to running without segmenting respondents into groups did not find significant differences in the items of the running-commitment scale according to age [15,25]. In other studies identifying groups according to their motivations for running, age was found to be a variable that differentiated the identified clusters [27,29,30]. The results varied according to gender, with some studies showing differences in the commitment to running according to this variable [10,19,21,25,37], while other studies, in the same vein as the results of this study, detected no significant differences by gender [15]. It is important to note that the profile of the runners in these studies was runners of long-distance races such as half-marathons and marathons, different from the sample analysed in this study. Regarding the level of studies, in the work of Zarauz and Ruiz-Juan [19] on marathon runners, it was observed that a lower level of studies significantly predicted the commitment to running. In this study, it was found that there were significant disproportions of runners with a higher level of studies among runners with a lower level of commitment to running. In any case, more studies on amateur short-distance runners are needed to determine which sociodemographic variables contribute to differentiating subgroups of runners according to their commitment to running.

On the other hand, variables related to sports habits (training frequency, preferred distance, years of running, number of kilometres run weekly, level at which runners considered themselves to be, participation in long-distance races, and membership of clubs and sports federations) also contributed to significantly differentiating the groups identified, confirming the need to consider runners from a heterogeneous point of view, as observed in other studies [27,29,30,61]. In previous studies, it was found that weekly training frequency, number of years of running, and number of kilometres run were positively related to commitment to running [10,21,22,23,25]. Similarly, a preference for participating in long-distance running was associated with higher scores on commitment to running [25,37,62].

From the point of view of the variables related to the running-addiction scale, it is worth noting that statistically significant differences were found between the groups, proving the existence of a higher level of involvement in the activity in runners with a high commitment to running. In the theoretical framework, it has been emphasized that previous studies have proved that there is a high correlation between higher values of commitment to running and addiction to this physical activity, although both constructs may be influenced by different variables. In a previous study, it was observed that, in half-marathon runners, the variable that seemed to have most importance with respect to why a half-marathon runner goes from being healthily committed to his sport practice to being pathologically addicted to it is the number of kilometres he runs each week: if it is low, a healthy commitment increases, while if it is high, in men, negative addiction increases [10]. In the present work, although this finding was not empirically proven, it is observed that the runners in the highly committed group train significantly more kilometres per week than the other groups and, in addition, they show a significantly higher tendency to agree on statements related to running addiction, such as the item stating that, some days, even if they do not feel like running, they practice the activity anyway. In this sense, the points made by Ruiz-Juan, Zarauz, and Flores-Allende [10] could be confirmed but in runners who participated in urban popular races of reduced distance. In any case, more empirical evidence is needed to confirm the hypothesized relationship between the number of kilometres run and running addiction in amateur short-distance runners.

On the other hand, analysis of the groups’ profile verified that the group denominated as highly committed presents a tendency to agree on most of the indicators related to the factor of enthusiasm to run, indicating a clear commitment through the practice of this activity. In contrast, on the aspects related to the affliction factor from running, they show the inverse tendency, which also corroborates the high commitment of these runners to this physical activity. This is the group with the highest proportion of people with the lowest level of studies, in contrast to the group with the lowest commitment to running, indicating a similar trend to that observed by Zarauz and Ruiz-Juan [19] on the influence of this variable on the commitment to running. This is a group with sporting habits defined by the constant and regular practice of this physical activity: they tend to run alone, have a training frequency of three or more times per week, display a preference for distances in popular races between 7.5 km and 15 km, are members of sports clubs, have the highest average number of kilometres run weekly, have the greatest number of years practising running, and have the highest average number of long-distance races run. This type of runner has been identified by Leedy [37] as “committed runners”. Additionally, Carmack and Martens [21] related the high commitment to running to running addiction; these runners were later classed by Pargman [20] as addict-dependent. However, this concept should be considered in a positive sense, that is, as defined by this author, from the perspective that they are participants who show pleasure, enjoyment, and joy in the practice of this sport, as identified through the average scores observed on the indicators of the running enthusiasm factor.

With respect to the group known as moderately committed, a more neutral and moderate attitude is observed in the indicators referring to the enthusiasm for running, highlighting aspects such as the desire to run or pleasure in running while showing a neutral evaluation on items such as the statement that the practice of this physical activity is the high point of the day or of vital importance for their lives. In the case of items on the affliction from running factor, they present a tendency towards disagreement, albeit less pronounced than that of the group with greater commitment. Their sociodemographic profile differs in that they are older than the rest of the groups and have a higher level of education than the most committed group. The sports habits of this group present intermediate levels between the groups with lesser commitment and greater commitment, with the highlights being that they tend to run alone, their training frequency is three to five times per week, they display a preference for short-distance races (less than 10 km), they run a shorter distance each weekly than the highly committed group, and they have fewer years practising this physical activity. On the other hand, regarding the indicators on the running addiction scale, they do not agree that they need to run at least once every day and show a slight tendency towards agreement on the item regarding having stopped running for reasons other than an injury. This runner profile is characterized by a recreational type of orientation and less commitment to the practice of running despite considering it an enjoyable and positive experience for their lives. Previous work on motivation and commitment to running has identified profiles of recreational runners [37], relational runners [5], socializing hedonists [27], and social competitors [29], who may have similarities in terms of their sporting habits and sociodemographic profile. This group could be identified with a healthier profile from a psychological point of view, since they do not show symptoms of possible running addiction, as could be deduced from the scores of the indicators extracted from the running addiction scale.

Finally, the group dubbed slightly committed is the group with the lowest level of commitment to running because it is the group that disagrees most with the indicators related to enthusiasm for running (running is of vital importance to their life; the practice of enriches their life; they reorganize schedules in their daily life; and running is the highlight of the day). Unlike for the other two groups, the only indicator with a positive trend for this group is the fact that they consider running a pleasant activity. In the same way, the other groups disagree with the aspects related to affliction from running. However, the slightly committed group has a certain tendency towards low motivation and considers the activity to be drudgery, as the tendency towards neutrality observed in some scores of the running affliction factor points out. From the point of view of sociodemographic characteristics, this is the youngest group and has the highest level of studies. They are runners who train less frequently (once or twice a week), prefer short-distance races (less than 7.5 km), are less likely to have affiliations with sports clubs, run fewer kilometres weekly, and have fewer years practising this sport. There is a lower degree of dependence on the activity, as seen from the scores on the running-addiction indicators, giving a secondary role to the practice of running.

### 4.1. Practical Implications

The objective of this research was to analyze the commitment of runners among urban runners and to segment and classify the runners. Research in this area and on commitment to running is limited, especially among urban short-distance runners. The groups of runners were identified and described sociodemographic profile and sports habits: highly committed, moderately committed, and slightly committed. The conclusions of the work can be useful for the administration, management, and organization of local sports in terms of knowing the profiles of participants in popular races and of developing measures to improve the quality of life of the participants. In this regard, a high commitment to running can imply risks for health and can devolve into addictive behaviours that must be prevented to ensure that a runner is healthily committed to their sport practice rather than pathologically addicted to it. Running is an activity that has experienced an important boom in Spanish cities such as Valencia, requiring the implementation of sports policies and strategic plans that respond to the social demands of this collective. For this reason, it is advisable to offer information and public services (sports, health, psychology, and education) to runners through the organizations and communication initiatives associated with this popular activity. Additionally, the identification of different profiles, characteristics, and habits can benefit sport entities and public organizations to encourage sport activity.

### 4.2. Limitations and Future Lines of Research

This work presents some limitations that should be taken into account when generalizing and extrapolating the results to other populations; for example, the type of sampling used does not allow for generalization of the results to the set of urban amateur runners. In carrying out f more studies of different populations of amateur runners, efforts should be made to identify subgroups of the population of urban runners in different countries and regions with the aim of elaborating typologies of runners at the international level as well as identifying possible differences according to sociocultural and socioeconomic characteristics. Similarly, the relationship between commitment to running and possible addictions to this physical activity in runners who are starting out in the sport or who participate in shorter-distance races should be explored in greater depth with the aim of preventing future behaviour that could be dangerous to health.

## 5. Conclusions

The aim of this paper is to analyse the commitment to running among urban runners by identifying groups with a greater or lesser commitment to this sport. Three groups of urban amateur runners with different levels of commitment to running were identified: highly committed, moderately committed, and slightly committed. Also, the study identifies two factors within the psychological construct of commitment to running: enthusiasm for running and affliction from running.

Sociodemographic variables related to age and educational level contribute to differentiating the groups of amateur runners according to their commitment to running. It is also found that most of the variables related to sports habits contribute to differentiating the groups: the frequency with which they go out to run, their preferred distance in popular races, the number of years they have practised running, the number of kilometres run weekly, the level they consider themselves to be at as runners, participation in long-distance races, and membership in clubs and sports federations.

Highly committed runners positively value the aspects related to enthusiasm for running and show the reverse trend in the aspects related to the affliction from running.

Moderately committed runners are characterized by a moderate attitude towards running enthusiasm and a tendency to disagree on aspects related to running affliction.

Slightly committed runners show a low commitment to the practice of running, with a tendency towards disagreement on the indicators related to enthusiasm for running except the one related to considering it a pleasant activity. They disagree with the aspects related to the affliction from running, although with a tendency to neutrality when considering running to be a drudgery and an activity for which they have low motivation.

## Figures and Tables

**Table 1:** Sociodemographic characteristics of the sample.

Variable	Response Option	Mean and Percentage
Age		39.48 (SD ^1^ = 9.21)
Gender	Male	74.4%
	Female	25.6%
Occupation	Employee	88.4%
	Unemployed	6.1%
	Student	1.2%
	Other (retired, pensioner, domestic tasks, etc.)	4.3%
Level of Studies	Primary	6.1%
	Secondary	6.2%
	Baccalaureate/ Professional training	28.5%
	University	59.2%
Income Level	Less than 12,000 euros	27.0%
	12,001–18,000 euros per year	24.3%
	18,001–24,000 euros per year	18.2%
	24,001–30,000 euros per year	14.2%
	30,001–36,000 euros per year	6.9%
	More than 36,001 euros per year	9.5%

^1^ SD = Standard Deviation.

**Table 2:** Mean, standard deviation, corrected item–total correlation, alpha if the item is removed, asymmetry, and kurtosis values of the indicators of the running-commitment scale.

Number	Items	Means (SD) ^1^	R IT-c ^2^	α without Item	Asymmetry	Kurtosis
CR1	I am looking forward to running	3.79 (0.93)	0.62	0.79	−0.58	0.21
CR2	Running is drudgery (R)	3.83 (1.12)	0.53	0.80	−0.68	−0.46
CR3	I do not enjoy running (R)	4.34 (0.91)	0.48	0.80	−1.54	2.21
CR4	Running is vitally important to me	3.23 (1.10)	0.59	0.79	−0.23	−0.51
CR5	Life is so much richer as a result of running	3.41 (1.07)	0.56	0.80	−0.47	−0.26
CR6	Running is pleasant	4.09 (0.75)	0.56	0.80	−0.80	1.39
CR7	I dread the thought of running (R)	4.72 (0.68)	0.30	0.82	−3.01	10.20
CR8	I would arrange or change my schedule to meet my need to run	3.29 (1.19)	0.46	0.81	−0.37	−0.68
CR9	I have to force myself to run (R)	3.66 (1.12)	0.42	0.81	−0.46	−0.63
CR10	To miss a day’s run is a sheer relief (R)	4.41 (0.83)	0.39	0.81	−1.45	1.89
CR11	Running is the high point of my day	2.93 (1.15)	0.47	0.81	−0.09	−0.68

^1^ SD = Standard Deviation; ^2^ Corrected item–total correlation; (R) = Reverse scoring; 1 = Totally disagree, 2 = Disagree, 3 = Neither agree nor disagree, 4 = Agree, and 5 = Totally agree.

**Table 3:** Rotated factor structure of the commitment-to-running scale of runners participating in popular endurance races, communalities, eigenvalues, and explained variance.

Number	Items	F1	F2	Com. ^1^
	*Factor 1: Enthusiasm for running*			
CR1	I am looking forward to running	0.56		0.48
CR4	Running is vitally important to me	0.81		0.64
CR5	Life is so much richer as a result of running	0.78		0.59
CR6	Running is pleasant	0.40		0.37
CR8	I would arrange or change my schedule to meet my need to run	0.62		0.37
CR11	Running is the high point of my day	0.65		0.41
	*Factor 2: Affliction from running*			
CR2	Running is drudgery		0.57	0.43
CR3	I do not enjoy running		0.67	0.48
CR7	I dread the thought of running		0.67	0.40
CR9	I have to force myself to run		0.56	0.34
CR10	To miss a day’s run is a sheer relief		0.67	0.43
G-H Index		0.85	0.80	
Eigenvalue		4.01	2.01	
Variance Explained (%)		36.51	18.28	
Items		6	5	

^1^ Com. = Communality.

**Table 4:** Factorial loads, Cronbach’s alpha, composite reliability, and average variance extracted values from commitment to running scale indicators.

Number	Items	λ	α	CR ^1^	AVE ^2^
	*Factor 1: Enthusiasm for running*		0.82	0.83	0.45
CR1	I am looking forward to running	0.68			
CR4	Running is vitally important to me	0.79			
CR5	Life is so much richer as a result of running	0.76			
CR6	Running is pleasant	0.54			
CR8	I would arrange or change my schedule to meet my need to run	0.61			
CR11	Running is the high point of my day	0.62			
	*Factor 2: Affliction from running*		0.76	0.77	0.40
CR2	Running is drudgery	0.66			
CR3	I do not enjoy running	0.69			
CR7	I dread the thought of running	0.57			
CR9	I have to force myself to run	0.6			
CR10	To miss a day’s run is a sheer relief	0.64			

^1^ CR = Composite Reliability; ^2^ AVE = Average Variance Extracted.

**Table 5:** Average scores for each variable in the three clusters (obtained through the k-averages method).

Number	Items	1 = Highly Committed (n = 650) (SD) ^1^	2 = Moderately Committed (n = 749) (SD) ^1^	3 = Lowly Committed (n = 407) (SD) ^1^	F	*p*-Value
	*Factor 1: Enthusiasm for running*	4.15 (0.46)	3.41 (0.35)	2.43 (0.41)	2263.89	<0.001 *
CR1	I am looking forward to running	4.42 (0.64)	3.73 (0.73)	2.89 (0.87)	543.02	<0.001 *
CR4	Running is vitally important to me	4.08 (0.78)	3.16 (0.77)	2.00 (0.80)	896.45	<0.001 *
CR5	Life is so much richer as a result of running	4.12 (0.78)	3.47 (0.74)	2.18 (0.87)	768.53	<0.001 *
CR6	Running is pleasant	4.51 (0.58)	4.01 (0.59)	3.58 (0.86)	263.28	<0.001 *
CR8	I would arrange or change my schedule to meet my need to run	4.09 (0.91)	3.26 (0.93)	2.09 (0.97)	577.43	<0.001 *
CR11	Running is the high point of my day	3.71 (0.96)	2.83 (0.90)	1.87 (0.88)	516.87	<0.001 *
	*Factor 2: Affliction from running*	1.32 (0.32)	2.04 (0.65)	2.16 (0.68)	388.42	<0.001 *
CR2	Running is drudgery	1.46 (0.75)	2.39 (1.01)	2.91 (1.14)	320.47	<0.001 *
CR3	I do not enjoy running	1.16 (0.54)	1.89 (0.96)	2.04 (0.93)	192.12	<0.001 *
CR7	I dread the thought of running	1.06 (0.33)	1.46 (0.85)	1.31 (0.65)	66.28	<0.001 *
CR9	I have to force myself to run	1.73 (0.89)	2.65 (1.03)	2.77 (1.16)	190.17	<0.001 *
CR10	To miss a day’s run is a sheer relief	1.20 (0.52)	1.82 (0.89)	1.79 (1.90)	126.35	<0.001 *

^1^ SD = Standard Deviation; * Statistically significant mean differences *p* < 0.001.

**Table 6:** Characteristics of the different groups (clusters).

Variable	Response Option	1 = Highly Committed (n = 650)	2 = Moderately Committed (n = 749)	3 = Lowly Committed (n = 407)
Age * F(2, 1803) = 5.73, *p* = 0.03		39.51 (SD ^1^ = 9.33)	40.13 (SD ^1^ = 9.26)	38.22 (SD ^1^ = 8.79)
Gender χ^2^(2) = 2.71, *p* = 0.26	Male	76.62%	73.03%	73.22%
	Female	23.38%	26.97%	26.78%
Occupation χ^2^(6) = 2.13, *p* = 0.91	Employee	88.15%	88.79%	87.96%
	Unemployed	6.92%	5.47%	5.90%
	Student	1.08%	1.34%	1.23%
	Other (retired, pensioner, domestic tasks, etc.)	3.85%	4.41%	4.91%
Level of studies ** χ^2^(6) =18.94, *p* = 0.004 C ^2^ = 0.10	Primary	8.46% ^(3)^	5.47%	3.44%
	Secondary	7.23%	6.14%	4.67%
	Baccalaureate/Professional training	29.85%	27.64%	27.76%
	University	54.46%	60.75%	64.13% ^(1)^
Income level χ^2^(10) = 13.71, *p* = 0.19	Less than 12,000 euros	29.38%	25.90%	25.06%
	12,001–18,000 euros per year	25.85%	22.56%	24.82%
	18,001–24,000 euros per year	15.54%	19.49%	20.15%
	24,001–30,000 euros per year	14.00%	14.69%	13.51%
	30,001–36,000 euros per year	7.23%	6.14%	7.86%
	More than 36,001 euros per year	8.00%	11.21%	8.60%
How often you run during the week? *** χ^2^(6) = 290.78, *p* ≤ 0.001 C ^2^ = 0.37	Five or more times a week	14.31% ^(2) (3)^	4.01%	1.97%
	Three to five times a week	62.46% ^(2) (3)^	51.27% ^(3)^	29.73%
	Once or twice a week	21.69%	40.32% ^(1)^	51.84% ^(1) (2)^
	Less frequently	1.54%	4.41% ^(1)^	16.46% ^(1) (2)^
Preferred distance in popular races *** χ^2^(10) =119.49, *p* ≤ 0.001 C ^2^ = 0.25	Less than 7.5 km	25.23%	36.85% ^(1)^	52.58% ^(1) (2)^
	Between 7.5 km and 10 km	28.15%	30.31%	27.27%
	Between 10 km and 15 km	27.69% ^(3)^	22.83% ^(3)^	13.51%
	Between 15 km and 20 km	10.15% ^(2) (3)^	6.14%	4.91%
	Between 20 km and 30 km	4.62% ^(3)^	2.54%	1.72%
	More than 30 km	4.15% ^(2)^	1.34%	0.00%
How do you usually run? χ^2^(2) = 0.34, *p* = 0.84	Alone	62.46%	61.15%	60.93%
	Accompanied	37.54%	38.85%	39.07%
Level you consider you are as a runner *** χ^2^(4) = 133.61, *p ≤* 0.001 C ^2^ = 0.26	High level	8.31% ^(2) (3)^	2.67%	1.47%
	Intermediate	64.92% ^(2) (3)^	53.14% ^(3)^	39.07%
	Low level	26.77%	44.19% ^(1)^	59.46% ^(2) (3)^
Are you a member of a sports club? χ^2^(2) = 53.84, *p* ≤ 0.001 C ^2^ = 0.17	Yes	48.77% ^(2) (3)^	40.05% ^(3)^	26.04%
	No	51.23%	59.95% ^(1)^	73.96% ^(2) (3)^
Are you sports federated? χ^2^(2) = 10.47, *p* = 0.005 C ^2^ = 0.08	Yes	6.92% ^(2) (3)^	3.60%	3.44%
	No	93.08%	96.40% ^(1)^	96.56% ^(1)^
Distance usually run weekly (kilometres) *** F(2, 1803) = 106.76, *p* ≤ 0.001		35.11 (SD ^1^ = 18.54)	26.16 (SD ^1^ = 14.65)	20.58 (SD ^1^ = 16.01)
Years running ** F(2, 1803) = 7.20, *p* = 0.01		8.04 (SD ^1^ = 8.95)	7.32 (SD ^1^ = 8.20)	6.08 (SD ^1^ = 6.68)
Participation in half marathons *** F(2, 1803) = 20.70, *p* ≤ 0.001		7.99 (SD ^1^ = 22.17)	4.05 (SD ^1^ = 10.06)	2.30 (SD ^1^ = 6.33)
Participation in marathons *** F(2, 1803) = 17.84, *p* ≤ 0.001		1.38 (SD ^1^ = 3.77)	0.74 (SD ^1^ = 2.74)	0.32 (SD ^1^ = 1.26)
Some days, even if I do not feel like running, I do it anyway *** F(2, 1803) = 104.78, *p* ≤ 0.001		3.71 (SD ^1^ = 1.02)	3.35 (SD ^1^ = 0.99)	2.76 (SD ^1^ = 1.13)
I feel like I need to run at least once every day *** F(2, 1803) = 150.05, *p* ≤ 0.001		3.04 (SD ^1^ = 1.20)	2.49 (SD ^1^ = 1.07)	1.84 (SD ^1^ = 0.97)
I have stopped running for at least a week for another reason that was not an injury *** F(2, 1803) = 46.84, *p* ≤ 0.001		3.04 (SD ^1^ = 1.44)	3.58 (SD ^1^ = 1.17)	3.75 (SD ^1^ = 1.31)
Future intentions regarding participation in urban popular races	I am willing to continue participating in popular urban races F(2, 1803) = 0.85, *p* = 0.45	4.42 (SD ^1^ = 1.03)	4.38 (SD ^1^ = 0.99)	4.35 (SD ^1^ = 0.91)
	I will recommend participation in popular urban races to others ** F(2, 1803) = 4.72, *p* = 0.009	4.48 (SD ^1^ = 3.77)	4.37 (SD ^1^ = 3.77)	4.32 (SD ^1^ = 3.77)
	I will speak well of popular urban races to others F(2, 1803) = 2.04, *p* = 0.13	4.46 (SD ^1^ = 0.89)	4.39 (SD ^1^ = 0.86)	4.37 (SD ^1^ = 0.80)

^1^ SD = Standard Deviation; ^2^ C = Contingency Coefficient; indications of statistically significant relationship or statistically significant mean differences: * *p* < 0.05; ** *p* ≤ 0 0.01; *** *p* ≤ 0.001; ^(1) (2) (3)^ results are based on bilateral tests with a level of significance 0.05. The results table shows for each significant pair the key of the group of runners with the proportion of the smallest column below the group of runners with the largest proportion of the column.
